# 1296. Effect of Preoperative Antibiotic Therapy on Operative Culture Yield for Diagnosis of Native Joint Septic Arthritis

**DOI:** 10.1093/ofid/ofad500.1135

**Published:** 2023-11-27

**Authors:** Ryan B Khodadadi, Pansachee Damronglerd, Jack W McHugh, Said El Zein, Brian Lahr, Brandon J Yuan, Omar M Abu Saleh, Gina A Suh, Aaron J Tande

**Affiliations:** Mayo Clinic, Rochester, Minnesota; Faculty of Medicine Thammasat University, Rochester, Minnesota; Mayo Clinic, Rochester, Minnesota; Mayo Clinic, Rochester, Minnesota; Mayo Clinic, Rochester, Minnesota; Mayo Clinic Rochester, Rochester, Minnesota; Mayo Clinic Rochester, Rochester, Minnesota; Mayo Clinic, Rochester, Minnesota; Mayo Clinic, Rochester, Minnesota

## Abstract

**Background:**

Native joint septic arthritis (NJSA) is diagnosed by synovial fluid analysis to evaluate for evidence of infection but may be definitively established by a positive gram stain or culture. Antibiotics given prior to synovial fluid sampling have been described to alter cell count, gram stain, and culture results and are conventionally deferred until after arthrocentesis to optimize diagnostic yield. However, there is limited data on the impact of preoperative antibiotic therapy on operative culture yield in the diagnosis of NJSA.

**Methods:**

Following IRB approval, we reviewed medical records of adults diagnosed with NJSA who underwent surgical intervention at Mayo Clinic facilities from January 2012 to December 2021. The effect of preoperative antibiotic therapy on operative culture yield was evaluated through a paired analysis of preoperative and operative cultures results utilizing logistic regression along with generalized estimating equations (GEE) to account for correlations of multiple measurements on the same subject.

**Results:**

A total of 299 patients with NJSA of 321 joints with the accompanying characteristics who underwent surgery met study criteria (**Table 1**). Joint distribution, microbiology, and antibiotic administration are outlined in **Table 2**. Among those treated with preoperative antibiotics, there was a significant decrease in yield between preoperative and operative cultures (68.0% to 57.1%, p < .0001), whereas in patients without preoperative antibiotic exposure, there was an increase in yield (60.9% to 67.4%, p = 0.244; **Figure 1**). Logistic regression analysis revealed that preoperative antibiotic exposure was more likely to decrease operative culture yield as compared to non-exposure (change, -10.9% vs. +6.5%; p = .006). Secondary analyses restricted to individuals who received preoperative antibiotics revealed that an increasing number of doses and earlier administration of initial antibiotic therapy were associated with lower rates of operative culture yield (**Figure 2**).

**Figure:**
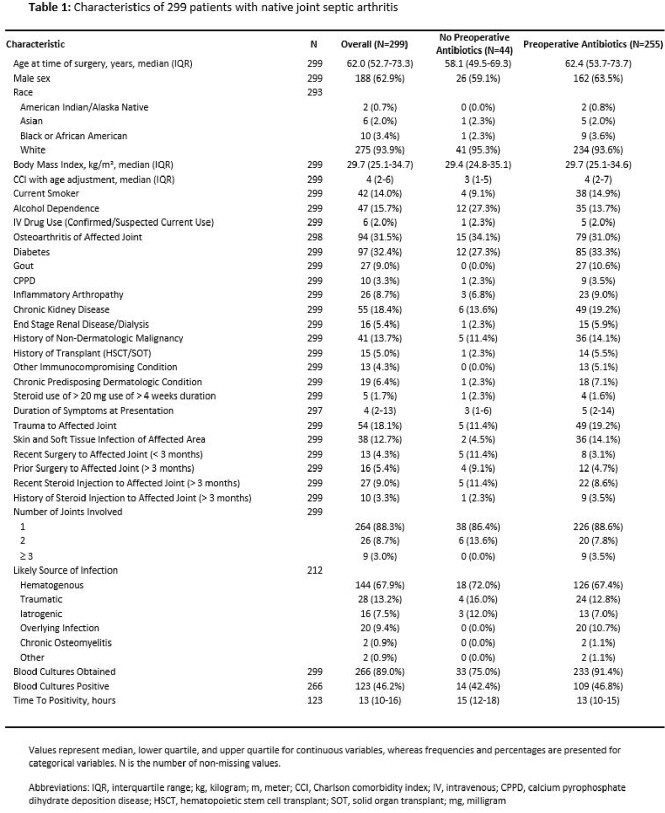

**Figure:**
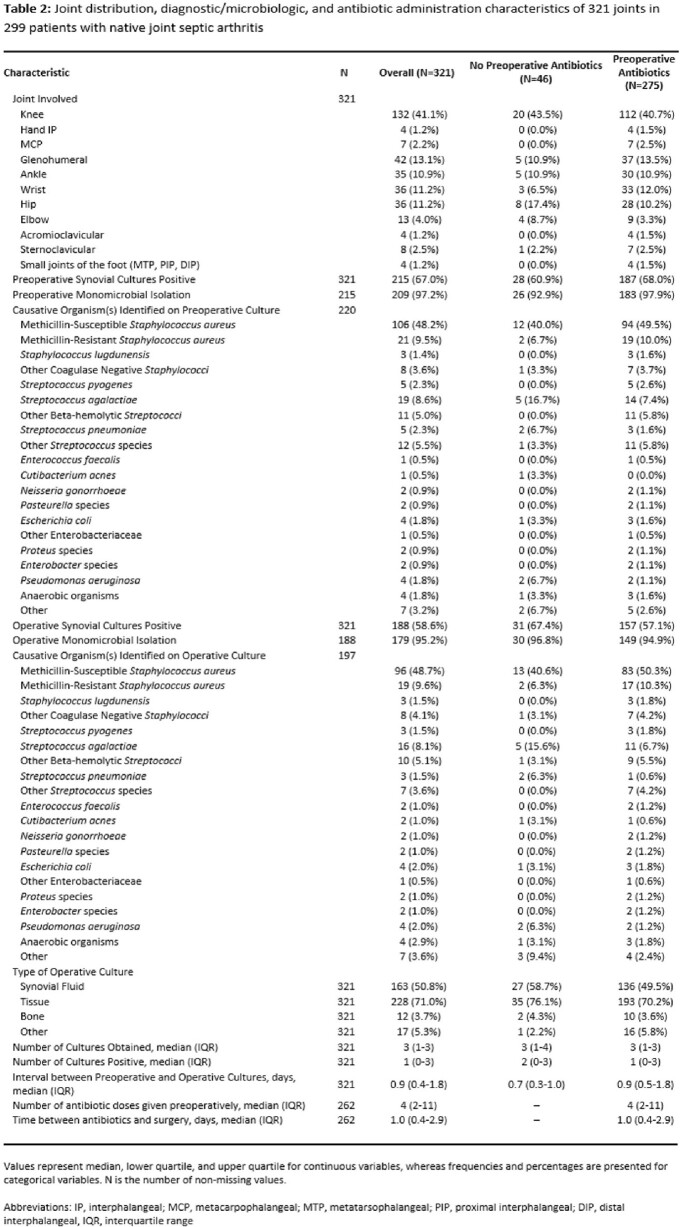

Joint distribution, diagnostic/microbiologic, and antibiotic administration characteristics of 321 joints in 299 patients with native joint septic arthritis

Composite Image 1: Figure 1, 2a/2b

**Figure:**
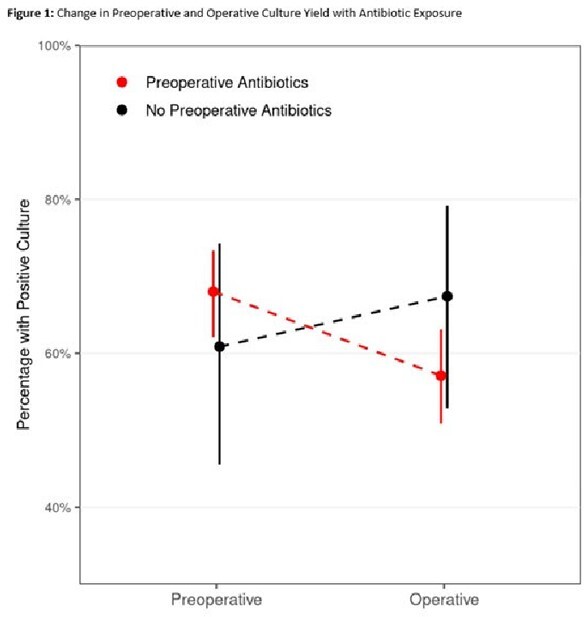

**Figure:**
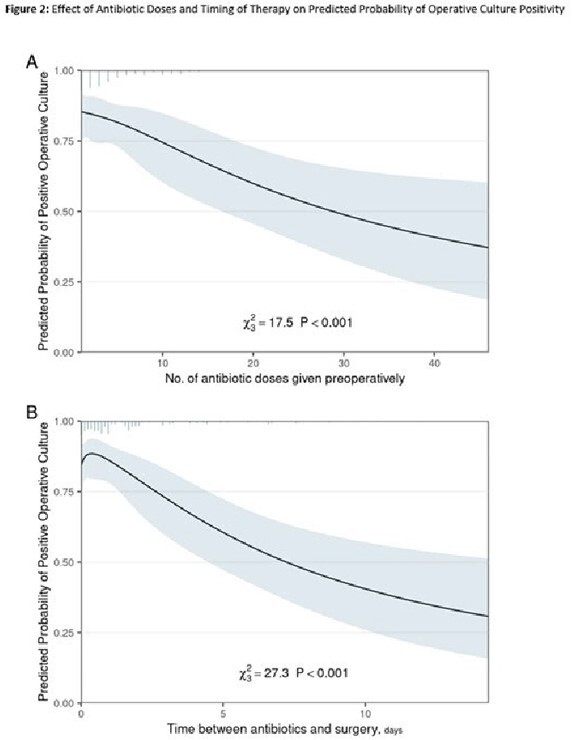

**Figure 1:** Change in Preoperative and Operative Culture Yield with Antibiotic Exposure Figure 2: Effect of Antibiotic Doses and Timing of Therapy on Predicted Probability of Operative Culture Positivity

**Conclusion:**

In patients with NJSA, preoperative antibiotic exposure resulted in a significant decrease in microbiologic yield of operative cultures as compared to patients in which antibiotic therapy was held prior to obtaining operative cultures.

**Disclosures:**

**Brandon J. Yuan, M.D.**, American Association of Orthopaedic Surgeons: Board Member|DePuy, A Johnson & Johnson Company: Advisor/Consultant|Mid America Orthopaedic Association: Board Member|Stryker: Advisor/Consultant **Gina A. Suh, M.D.**, Adaptive Phage Therapeutics: Grant/Research Support|Adaptive Phage Therapeutics: IP royalties|Phagelux: Grant/Research Support **Aaron J. Tande, M.D.**, Musculoskeletal Infection Society: Board Member|Wolters Kluwer Health - Lippincott Williams & Wilkins: Publishing royalties, financial or material support

